# Tonsil Slitting

**Published:** 1889-12-07

**Authors:** 


### SURGERY.

### TONSIL SLITTING.

This is the name given by Dr. Moritz Schmidt, of Wiesbaden, to an operation which he strongly advises in certain cases. It is now generally admitted that true abscesses of the tonsils are of rare occurrence, and that the pseudo-abscesses so commonly observed are merely the result of the distension of the follicles with their own secretion rendered unnaturally dense by the proliferation of the epithelial cells, which then undergo a process of caseation. The contents then liquefy from within, ultimately expelling the harder mass that hitherto had obstructed the orifice. In such cases "lancing," though desired by the patient, is unnecessary or hurtful. But in chronic cases, Dr. Schmidt believes that the retention of masses of cheesy and calcareous products of degeneration give rise to great inconvenience, alteration of the voice, sensations as of foreign bodies in the "throat," trigeminal neuralgia, etc., besides rendering the pharynx more vulnerable to diphtheritic or septic infection, and advises the thorough evacuation of the accumulated masses by plunging a strabismus knife deep into the tonsil, pressing it downwards, and then working it upwards and forwards, so as to completely divide the organ, when the upper half, which in such cases may protrude into the angle between the jaws, will often be found stuffed with concretions, the discharge of which gives speedy and entire relief. His remarks apply only to chronic cases, acute ones, as we have said, ending in spontaneous evacuation in a few days, and not calling for any operative interference.

